# Trends in incidence and mortality of nasopharyngeal cancer in China (2004–2018): an age-period-cohort analysis

**DOI:** 10.3389/fonc.2025.1592217

**Published:** 2025-07-08

**Authors:** Xu Chen, Wei Xia, Zhi-Hui Xu, Ya-Xi Suo, Long Xie

**Affiliations:** ^1^ State Key Laboratory of Oral & Maxillofacial Reconstruction and Regeneration, Key Laboratory of Oral Biomedicine Ministry of Education, Hubei Key Laboratory of Stomatology, School & Hospital of Stomatology, Wuhan University, Wuhan, Hubei, China; ^2^ Department of Paediatric Dentistry, School & Hospital of Stomatology, Wuhan University, Wuhan, China; ^3^ Department of Otolaryngology Head and Neck Surgery, Renmin Hospital of Wuhan University, Wuhan, China

**Keywords:** average annual percent change, age-period-cohort, incidence, mortality, nasopharynx cancer

## Abstract

**Objective:**

To analyze the trends in incidence and mortality of nasopharyngeal cancer (NPC) in China and its age-period-cohort effect, providing evidence for developing prevention and control strategies for NPC.

**Methods:**

NPC data were obtained from the Chinese Cancer Registry Annual Reports covering the period from 2004 to 2018. Joinpoint Regression Software (version 4.9.0.0) was used to calculate the Average Annual Percent Change (AAPC) and their corresponding 95% confidence intervals (CI) to assess the trends in incidence and mortality rates. We analyzed the age-period-cohort model of NPC in the Chinese population and estimated the effect of age, period, and cohort on NPC incidence and mortality.

**Results:**

The incidence and mortality rates of NPC show a steady declining trend. The age-specific incidence curves for NPC in Chinese males and females both peaked in the 50–54 age group, while the age-specific mortality curves peaked in the 70–74 and 75–79 age groups for males and females, respectively. Using the 2009–2013 period as a reference, the period effect rate ratios (RR) for NPC incidence and mortality in males during 2004–2008 were both greater than 1 and higher than those in females. Additionally, the cohort effect RR values for overall NPC incidence and mortality in China showed downward trend.

**Conclusion:**

Our study confirmed the effectiveness of NPC prevention and control strategies in China from 2004 to 2018. However, it underscores the need for targeted interventions among high-risk populations to further reduce the incidence and mortality rates of NPC.

## Introduction

Nasopharyngeal carcinoma (NPC) is a malignant tumor arising from the epithelial lining of the nasopharynx, with distinct geographic and ethnic distribution patterns (1). Based on GLOBOCAN 2020 data, an estimated 133,354 new cases of NPC were reported globally, with an age-standardized incidence rate (ASIR) of 1.5 per 100,000 person-years. Additionally, NPC accounted for approximately 80,008 deaths worldwide, corresponding to an age-standardized mortality rate (ASMR) of 0.9 per 100,000 person-years. These figures represent 0.7% of all new cancer cases and 0.8% of cancer-related deaths globally (2). In China, NPC is relatively rare in northern regions but highly prevalent in provinces such as Guangdong, Guangxi, and Hunan (3). Despite declining trends in both global incidence and mortality rates over recent decades due to lifestyle and environmental changes, NPC remains a major burden in high-risk regions (1).

The etiology of NPC is multifactorial, involving a complex interplay between genetic, environmental, and viral factors. A strong association has been established between Epstein-Barr virus (EBV) infection and NPC development, with EBV serology being a critical diagnostic and prognostic marker. Genetic susceptibility, particularly in individuals with certain human leukocyte antigens (HLA) haplotypes, also plays an important role (4). Environmental factors such as high consumption of salted fish, exposure to nitrosamines, and occupational hazards like formaldehyde further increase NPC risk (5, 6). Additionally, lifestyle factors such as smoking and drinking patterns contribute to its pathogenesis (7). Understanding these risk factors is essential for identifying high-risk populations and implementing targeted preventive measures.

Many studies use the incidence and mortality data of NPC in China, calculated by large models from the GBD (Global Burden of Disease) database, for evaluation and predictive analysis of future disease burden (8, 9). However, compared to the data source of the GBD, the data provided by the Chinese Cancer Registry Annual Report (CCRAR) more accurately reflects the true disease burden of cancer in China, which collects statistical data on the health of residents across 31 provinces, autonomous regions, and municipalities in China and uses the ICD-10 International Classification of Diseases statistical standard (10). To the best of our knowledge, there are currently no reports using NPC data from CCRAR to explore changes in incidence and mortality rates across different age groups in China. This study aims to analyze the disease status of NPC among different age groups and genders in China from 2004 to 2018 using data from CCRAR. Additionally, it seeks to examine the long-term trends in incidence and mortality of the disease through an age-period-cohort model and explore the potential underlying influences (age effect, period effect, and cohort effect).

## Methods

### Data collection

National data of NPC [ICD 10 codes C11] from 2004 to 2018 were sourced from the CCRAR compiled by the Tumor Registration and Reporting System published in 2008-2023. The Tumor Registration and Reporting System itself was officially launched in 2002 with approval from the Chinese Ministry of Health and now includes numerous cancer surveillance sites across various cities. When new cancer cases or deaths caused by malignant tumors are identified at hospitals and health facilities at different levels, these are reported to the Tumor Prevention and Reporting System’s management agency. The national cancer registry annual report includes data from over 700 cancer registries and accounting for 37.22% of the national population in 2018 (10). We divided the population into 15 age groups, each spanning five years (15-19, 20-24, …, 80-85, 85+) and applied five-year intervals to period groups (2004–2008, 2009–2013, 2014–2018). Population estimates for China were derived from the United Nations Population Division’s World Population Prospects.

### Joinpoint regression model

Trends in NPC incidence and mortality were analyzed using the annual percentage change (APC) and average annual percentage change (AAPC) with corresponding 95% confidence intervals (CIs). The log-linear model can be expressed as: $\text{E}\left[\text{y|x}\right]={\text{e}}^{{\text{β}}_{0}+{\text{β}}_{1}\text{x}+{\text{δ}}_{1}{\left(\text{x}-{\text{τ}}_{1}\right)}^{+}}+\ldots +{\text{δ}}_{\text{k}}{\left(\text{x}-{\text{τ}}_{\text{k}}\right)}^{+}$ , where y represents disease prevalence or mortality rate, x denotes the calendar year, β_1_ is regression coefficient, k indicates the number of join-points, the τk are the unknown join-points and a^+^ = a for a > 0 and 0 otherwise. The model’s trends are typically characterized using Annual Percentage Change (APC) and Average Annual Percentage Change (AAPC). APC measures the rate of change within each segment defined by join-points and is calculated as: $APC=\left[\frac{y_{x+1}-y_{x}}{y_{x}}\right]*100\%=\left(e^{\beta_{1}}-1\right)*100\%$ . Meanwhile, AAPC provides a summary measure of the overall trend across the entire study period and is computed as: $\text{AAPC }=\left(e^{\sum^{}w_{i}\beta_{i}/\sum^{}w_{i}}-1\right)*100\%$ , where *w_i_* represents the weight for each segment (11). An increasing trend in the age-standardized rate was identified when both the AAPC or APC and the lower limit of their 95% confidence interval were above zero. Conversely, a decreasing trend was observed when the AAPC or APC and the upper bound of the 95% confidence interval were below zero.

### Age-period-cohort model

The age-period-cohort model is used to analyze the effects of age, period, and cohort on disease rates. People of different age living in different age periods have different life-style factors, which might affect their risk of cancer development (12). We used the online tool provided by the National Cancer Institute (https://analysistools.cancer.gov/apc/) to perform age-period-cohort analysis on the incidence and mortality rates of NPC. The input for the web-based APC tool includes age-specific event counts and corresponding person-years across time, structured as a matrix with paired columns of rates. The tool generates various model-based estimates, such as longitudinal and cross-sectional age-specific rates, period and cohort rate ratios that reflect the overall trend (net drift), and age-specific annual percent changes (local drifts). Users can enter data directly using a Microsoft Excel worksheet or upload it as a CSV (comma-separated values) file. The results generated by the model can be exported in multiple formats, including Excel and R-compatible files (13).

Statistical procedures were executed using open-source R software (version 4.1.3). P values less than 0.05 were considered statistically significant.

## Results

### The incidence and trends of NPC in cancer registry areas in China

**Figure 1:**
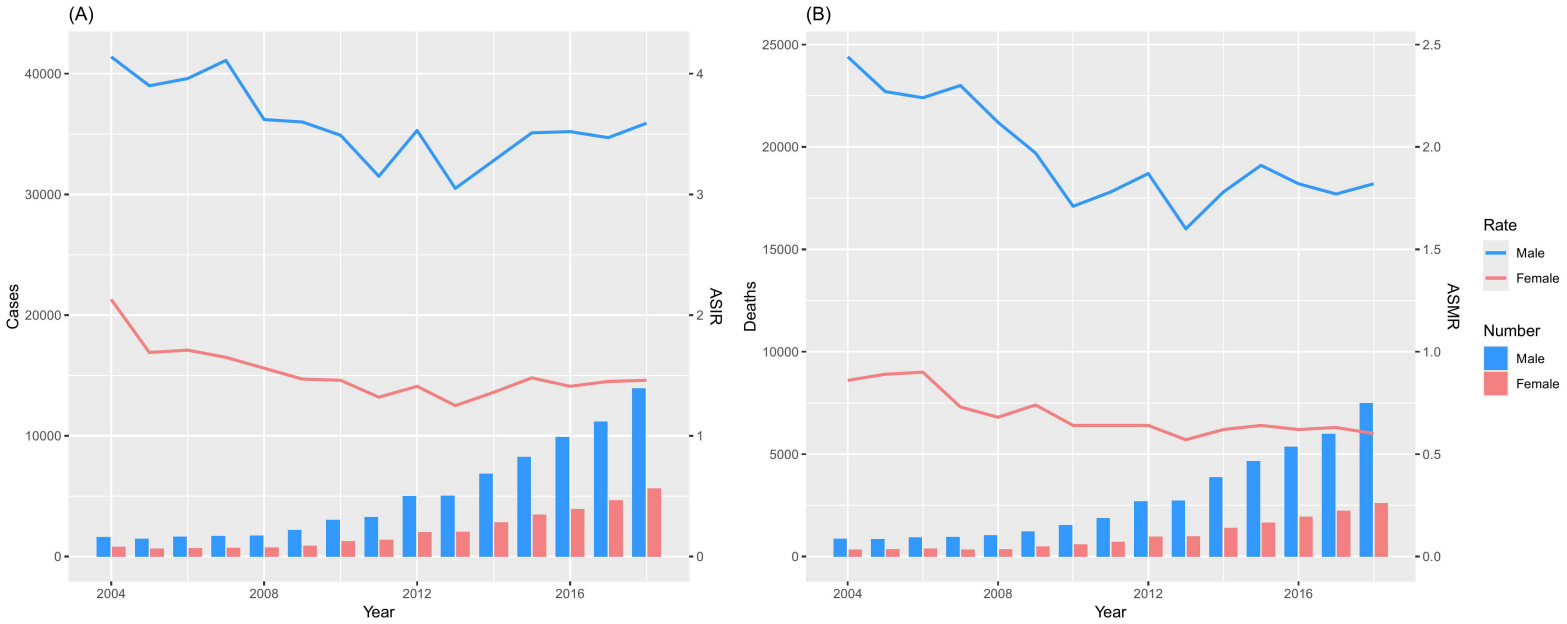
Cases and deaths of NPC from the Chinese Annual Cancer Registry, 2004-2018. **(A)** Incidence; **(B)** Death.

From 2004 to 2018, the number of newly diagnosed NPC cases among males in cancer registry areas in China increased from 1,598 to 13,912, representing a 7.71-fold rise. Similarly, the number of new cases among females rose from 809 to 5,652, showing a 7.99-fold increase (**Figure 1A**, **Table 1**). Despite the increase in case numbers, the ASIR for males decreased from 4.14 per 100,000 to 3.59 per 100,000, reflecting an 13.26% decline, with a corresponding AAPC of -1.26% (95% CI: -2.16 to -0.35). For females, the ASIR decreased from 2.13 per 100,000 to 1.46 per 100,000, marking a 31.46% decline, with an AAPC of -2.03% (95% CI: -3.22 to -0.82) (**Figure 1A**, **Table 1**). Both trends indicate a significant downward trajectory in NPC incidence rates over the study period.

**Table 1:** Burden and trends of NPC in the Chinese Cancer Registry in 2004 and 2018.

		2004		2018		Changes		
Measure	Sex	Number	ASR per 100,000	Number	ASR per 100,000	Change in absolute number (times)	Change in absolute rate (%)	2004–2018 AAPC of ASR (95%CI)
Incidence	Male	1,598	4.14	13,912	3.59	7.71	-13.26	-1.26 (-2.16 to -0.35)
	Female	809	2.13	5,652	1.46	7.99	-31.46	-2.03 (-3.22 to -0.82)
Death	Male	861	2.44	7,492	1.82	7.70	-25.41	-2.18 (-3.20 to -1.16)
	Female	329	0.86	2,607	0.60	6.92	-30.23	-2.74 (3.75 to -1.72)

ASR, age-standardized rate; CI, Confidence interval; AAPC, Average annual percent change; NPC, Nasopharyngeal Cancer.

### The mortality and trends of NPC in cancer registry areas in China

From 2004 to 2018, the number of NPC-related deaths among males in cancer registry areas in China increased from 861 to 7,492, representing a 7.70-fold rise. Similarly, the number of deaths among females rose from 329 to 2,607, showing a 6.92-fold increase (**Figure 1B**, **Table 1**). Despite the growth in absolute numbers, the ASMR for males decreased from 2.44 per 100,000 to 1.82 per 100,000, reflecting a 25.41% reduction, with a corresponding AAPC of -2.18% (95% CI: -3.65 to -0.69). For females, the ASMR decreased from 0.86 per 100,000 to 0.60 per 100,000, marking a 30.23% reduction, with an AAPC of -2.72% (95% CI: -4.30 to -1.11) (**Figure 1B**, **Table 1**). These results indicate a significant downward trend in NPC mortality rates during the study period.

### Trends in the age-specific incidence and mortality using joinpoint regression analysis

**Figure 2:**
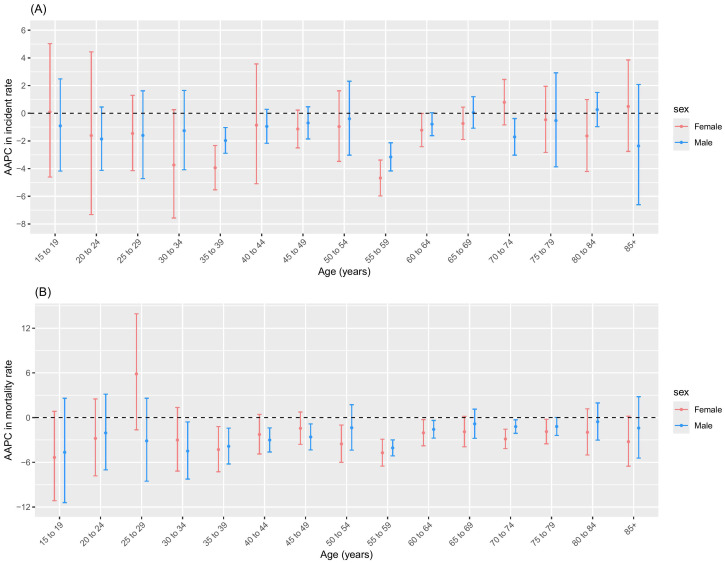
AAPC in incidence and mortality of age group over 15 years. **(A)** Incidence; **(B)** Mortality.

The incidence and mortality rates of NPC among males and females across all age groups showed an overall declining trend (**Table 2**, **Figure 2A**). From 2004 to 2018, in terms of NPC incidence rates, males showed a significant declining trend in the 35 to 39 and 70 to 74 age groups, while other age groups remained stable. For females, a significant decline was observed in the 35 to 39, 55 to 59, and 60 to 64 age groups, with a stable trend in other age groups. From 2004 to 2018, in terms of NPC mortality rates, males showed a significant declining trend in the 30 to 34, 35 to 39, 40 to 44, 45 to 49, 55 to 59, 60 to 64, and 70 to 74 age groups, while other age groups remained stable. For females, a significant decline was observed in the 35 to 39, 50 to 54, 55 to 59, 60 to 64, 70 to 74, and 75 to 79 age groups, with a stable trend in other age groups (**Table 2**, **Figure 2B**).

**Table 2:** AAPC and APC of the age-specific incidence and mortality of NPC in cancer registries.

Age	Incidence of male/female			Mortality of male/female		
	period	APC (%, 95% CI)	AAPC (%, 95% CI)	period	APC (%, 95% CI)	AAPC (%, 95% CI)
**15 to 19**	2004-2018	-0.91 (-4.18 to 2.48)	-0.91 (-4.18 to 2.48)	2004-2018	-4.66 (-11.41 to 2.60)	-4.66 (-11.41 to 2.60)
20 to 24	2004-2018	-1.86 (-4.13 to 0.45)	-1.86 (-4.13 to 0.45)	2004-2018	-2.06 (-7.01 to 3.15)	-2.06 (-7.01 to 3.15)
25 to 29	2004-2018		-1.60 (-4.72 to 1.62)	2004-2018		-3.13 (-8.53 to 2.59)
	2004-2009	-13.44 (-20.30 to -5.98)		2004-2008	-20.84 (-34.82 to -3.86)	
	2009-2018	5.66 (2.16 to 9.29)		2008-2018	5.02 (0.12 to 10.17)	
30 to 34	2004-2018	-1.26 (-4.08 to 1.65)	-1.26 (-4.08 to 1.65)	2004-2018		-4.49 (-8.25 to -0.58)
				2004-2010	-16.77 (-23.22 to -9.78)	
				2010-2018	5.89 (0.51 to 11.55)	
35 to 39	2004-2018	-1.97 (-2.89 to -1.03)	-1.97 (-2.89 to -1.03)	2004-2018	-3.85 (-6.22 to -1.42)	-3.85 (-6.22 to -1.42)
40 to 44	2004-2018	-0.95 (-2.17 to 0.28)	-0.95 (-2.17 to 0.28)	2004-2018	-3.01 (-4.62 to -1.38)	-3.01 (-4.62 to -1.38)
45 to 49	2004-2018	-0.70 (-1.86 to 0.47)	-0.70 (-1.86 to 0.47)	2004-2018	-2.60 (-4.33 to -0.84)	-2.60 (-4.33 to -0.84)
50 to 54	2004-2018		-0.40 (-3.03 to 2.31)	2004-2018		-1.36 (-4.36 to 1.73)
	2004-2013	-3.79 (-6.45 to -1.05)		2004-2013	-5.04 (-8.06 to -1.93)	
	2013-2018	6.02 (-1.04 to 13.57)		2013-2018	5.63 (-2.4 to 14.32)	
55 to 59	2004-2018	-3.16 (-4.17 to -2.13)	-3.16 (-4.17 to -2.13)	2004-2018	-4.07 (-5.14 to -2.99)	-4.07 (-5.14 to -2.99)
60 to 64	2004-2018	-0.79 (-1.62 to 0.04)	-0.79 (-1.62 to 0.04)	2004-2018	-1.58 (-2.75 to -0.39)	-1.58 (-2.75 to -0.39)
65 to 69	2004-2018	0.05 (-1.08 to 1.20)	0.05 (-1.08 to 1.20)	2004-2018		-0.84 (-2.78 to 1.14)
				2004-2010	-5.46 (-9.15 to -1.62)	
				2010-2018	2.77 (0.17 to 5.45)	
70 to 74	2004-2018	-1.71 (-3.03 to -0.38)	-1.71 (-3.03 to -0.38)	2004-2018	-1.21 (-2.12 to -0.30)	-1.21 (-2.12 to -0.30)
75 to 79	2004-2018		-0.53 (-3.87 to 2.92)	2004-2018	-1.20 (-2.40 to 0.01)	-1.20 (-2.40 to 0.01)
	2004-2006	13.73 (-8.00 to 40.60)				
	2006-2011	-6.65 (-12.70 to -0.17)				
	2011-2018	0.17 (-2.63 to 3.05)				
80 to 84	2004-2018	0.26 (-0.97 to 1.50)	0.26 (-0.97 to 1.50)	2004-2018		-0.56 (-3.02 to 1.97)
				2004-2006	15.24 (-4.53 to 39.11)	
				2006-2018	-2.97 (-4.05 to -1.89)	
85+	2004-2018	-2.36 (-6.61 to 2.08)	-2.36 (-6.61 to 2.08)	2004-2018	-1.40 (-5.43 to 2.80)	-1.40 (-5.43 to 2.80)
**15 to 19**	2004-2018	0.09 (-4.61 to 5.03)	0.09 (-4.61 to 5.03)	2004-2018		-5.35 (-11.15 to 0.83)
				2004-2016	1.73 (-1.08 to 4.63)	
				2016-2018	-38.61 (-61.79 to -1.39)	
20 to 24	2004-2018	-1.61 (-7.32 to 4.44)	-1.61 (-7.32 to 4.44)	2004-2018	-2.79 (-7.82 to 2.51)	-2.79 (-7.82 to 2.51)
25 to 29	2004-2018	-1.46 (-4.14 to 1.29)	-1.46 (-4.14 to 1.29)	2004-2018	5.86 (-1.63 to 13.93)	5.86 (-1.63 to 13.93)
30 to 34	2004-2018		-3.74 (-7.57 to 0.26)	2004-2018	-3.01 (-7.18 to 1.35)	-3.01 (-7.18 to 1.35)
	2004-2009	-16.82 (-25.07 to -7.67)				
	2009-2018	4.41 (0.05 to 8.95)				
35 to 39	2004-2018	-3.94 (-5.53 to -2.33)	-3.94 (-5.53 to -2.33)	2004-2018	-4.29 (-7.27 to -1.21)	-4.29 (-7.27 to -1.21)
40 to 44	2004-2018		-0.86 (-5.09 to 3.56)	2004-2018	-2.26 (-4.88 to 0.44)	-2.26 (-4.88 to 0.44)
	2004-2007	5.36 (-5.27 to 17.18)				
	2007-2010	-13.42 (-30 to 7.09)				
	2010-2018	1.96 (-0.38 to 4.35)				
45 to 49	2004-2018	-1.14 (-2.50 to 0.23)	-1.14 (-2.50 to 0.23)	2004-2018	-1.44 (-3.58 to 0.75)	-1.44 (-3.58 to 0.75)
50 to 54	2004-2018		-0.96 (-3.48 to 1.62)	2004-2018		-3.53 (-6.00 to -0.99)
	2004-2013	-5.21 (-7.73 to -2.62)		2004-2011	-9.33 (-13.04 to -5.46)	
	2013-2018	7.18 (0.33 to 14.50)		2011-2018	2.65 (-1.55 to 7.03)	
55 to 59	2004-2018	-4.68 (-5.97 to -3.38)	-4.68 (-5.97 to -3.38)	2004-2018	-4.72 (-6.50 to -2.91)	-4.72 (-6.50 to -2.91)
60 to 64	2004-2018	-1.22 (-2.41 to -0.01)	-1.22 (-2.41 to -0.01)	2004-2018	-2.04 (-3.78 to -0.26)	-2.04 (-3.78 to -0.26)
65 to 69	2004-2018	-0.74 (-1.90 to 0.44)	-0.74 (-1.90 to 0.44)	2004-2018	-1.90 (-3.91 to 0.16)	-1.90 (-3.91 to 0.16)
70 to 74	2004-2018	0.79 (-0.84 to 2.44)	0.79 (-0.84 to 2.44)	2004-2018	-2.87 (-4.16 to -1.57)	-2.87 (-4.16 to -1.57)
75 to 79	2004-2018	-0.47 (-2.83 to 1.94)	-0.47 (-2.83 to 1.94)	2004-2018	-1.88 (-3.51 to -0.22)	-1.88 (-3.51 to -0.22)
80 to 84	2004-2018	-1.64 (-4.21 to 0.99)	-1.64 (-4.21 to 0.99)	2004-2018	-1.97 (-5.02 to 1.18)	-1.97 (-5.02 to 1.18)
85+	2004-2018	0.49 (-2.76 to 3.85)	0.49 (-2.76 to 3.85)	2004-2018	-3.23 (-6.52 to 0.18)	-3.23 (-6.52 to 0.18)

APC, annual percent change; AAPC, Average annual percent change; CI, Confidence interval; NPC, Nasopharyngeal Cancer.

The bolded values represent age groups, where the first 15 to 19 corresponds to male data and the second 15 to 19 corresponds to female data.

From 2009 to 2018, the most pronounced increase in NPC incidence rates was observed in males aged 25–29 years and females aged 30–34 years, with APC values of 5.66% (2.16 to 9.29) and 4.41% (0.05 to 8.95), respectively (**Table 2**). The most pronounced increase in NPC mortality rates of males was observed in aged 25–29 years during 2008–2018 and aged 30–34 years during 2010-2018, with APC values of 5.02% (0.12 to 10.17) and 5.89% (0.51 to 11.55), respectively (**Table 2**).

### Age-period-cohort modeling in the incidence and mortality of NPC in China

**Figure 3:**
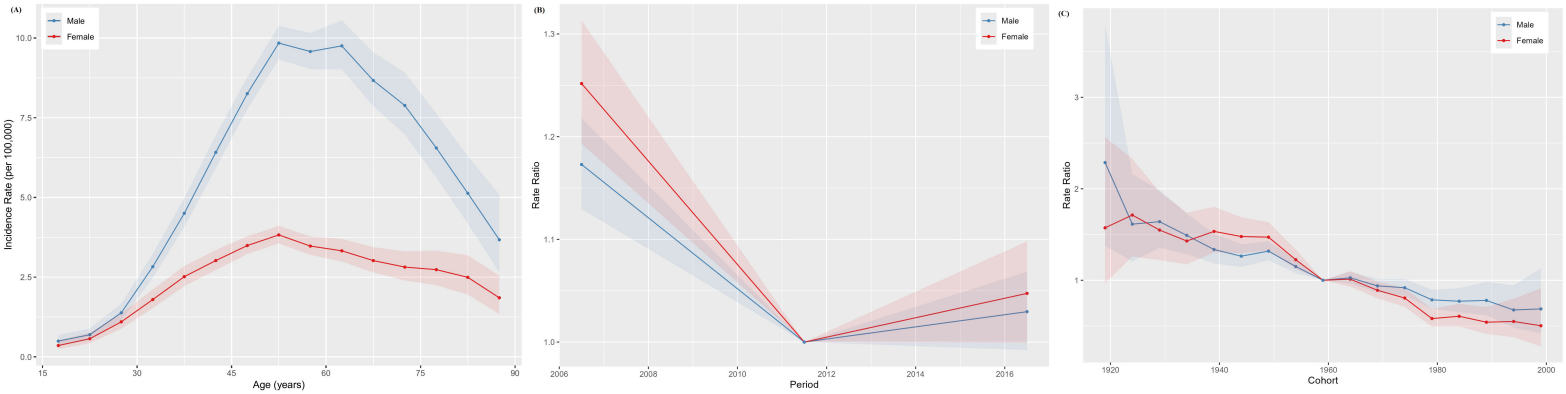
The age–period–cohort analysis of NPC incidence in China. **(A)** Longitudinal age curve; **(B)** Period rate ratio; **(C)** Cohort rate ratio.

**Figure 4:**
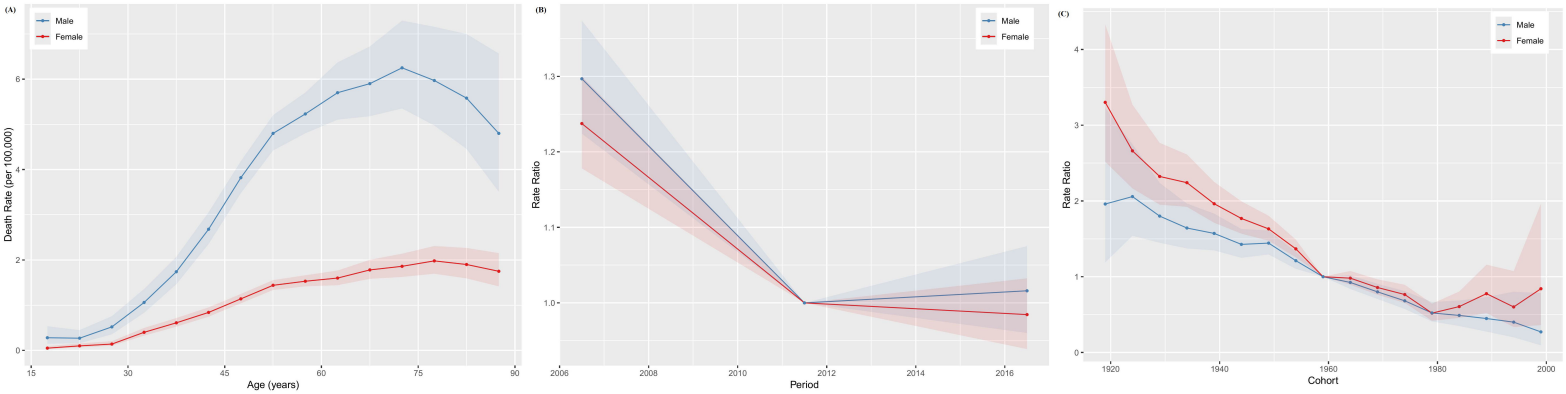
The age–period–cohort analysis of NPC death rate in China. **(A)** Longitudinal age curve; **(B)** Period rate ratio; **(C)** Cohort rate ratio.

The age-specific incidence curves for NPC in Chinese males and females both peaked in the 50–54 age group (**Figure 3A**), while the age-specific mortality curves peaked in the 70–74 and 75–79 age groups for males and females, respectively (**Figure 4A**). Additionally, both the incidence and mortality rates of NPC were higher in males compared to females (**Figures 3A**, **4A**). The period rate ratio for NPC incidence and mortality risk in males and females showed an initial decline, followed by a gradual stabilization (**Figures 3B**, **4B**). The cohort effect for both males and females exhibited a declining trend (**Figures 3C**, **4C**).

The Wald test validated the statistical significance of these trends and effects in the age-period-cohort model, confirming the robustness of the patterns observed in the age, period, and cohort effects, as detailed in **Supplementary Table S1**.

## Discussion

Our study provides a comprehensive analysis of the trends in incidence and mortality of NPC in China from 2004 to 2018. The results highlight a steady decline in both the ASIR and ASMR over the study period, reflecting the success of NPC prevention and control strategies in high-risk regions. Although the absolute number of new cases and deaths increased during this period, this rise may be partly attributed to population growth and aging, rather than a true increase in disease risk (8). Despite these promising trends, NPC continues to pose a significant public health challenge.

The observed decline in NPC incidence and mortality rates may be attributed to several factors. Foremost among these is the advancement in early detection and diagnosis, particularly through the use of EBV serology as a reliable biomarker (14). Improved public health awareness and the implementation of targeted screening programs have also played a significant role in identifying cases at earlier, more treatable stages (15). These developments have collectively contributed to better treatment outcomes and a notable reduction in NPC-related mortality. On one hand, lifestyle changes, including reduced consumption of salted fish and other nitrosamine-rich foods, may have decreased exposure to key environmental risk factors (16). The decline in the incidence of NPC can be attributed to several factors. On the other hand, stricter regulations on occupational hazards such as formaldehyde and the prevalence of current smokers have likely contributed to the decline in NPC incidence (17, 18). Since 2002, indoor formaldehyde concentrations in residential buildings, schools, and offices nationwide have been decreasing year by year (17). Additionally, improvements in air quality are also an important factor. From 1990 to 2017, the population-weighted annual average PM2.5 exposure in China decreased by 9%, and the ASMR attributable to air pollution in China declined by 66% (19). Compared with the monitoring results from 2014-2015, the current smoking rate has decreased, with a 2.8 percentage point decline in the overall population (18).

The increasing advancement of NPC treatment approaches has also contributed to the reduction in mortality. Radiotherapy and chemotherapy remain the main treatment modalities for NPC; however, their efficacy is limited in cases of locally advanced or metastatic tumors. Tumor immunotherapy, including vaccination, adoptive cell therapy, and immune checkpoint blockade, represents a promising therapeutic approach for NPC (20). Additionally, antiangiogenic therapy has also achieved remarkable results in the treatment of recurrent and metastatic nasopharyngeal carcinoma (21).

The observed higher incidence and mortality rates of NPC in males compared to females can be attributed to a combination of biological, lifestyle, and environmental factors. Males are more likely to be exposed to well-established risk factors for NPC, such as smoking and alcohol consumption, which are both associated with increased susceptibility to the disease (22, 23). Smoking is associated with higher mortality rates of NPC, and alcohol consumption is an important factor affecting the prognosis of NPC, with the adverse effects further amplified when combined with smoking (7, 24). Despite the overall decline in smoking rates in China, the prevalence of smoking remains significantly higher among males, which may partly explain their higher incidence and mortality rates (18). Moreover, males are more likely to work in occupations with exposure to hazardous chemicals, such as formaldehyde and wood dust, which are recognized as risk factors for NPC (5). These occupational exposures, coupled with lifestyle factors, create a cumulative risk that disproportionately affects males. Biological differences between males and females may also play a role. Hormonal factors, such as the protective effects of estrogen, have been hypothesized to reduce the risk of NPC in females (25). The female NPC patients have a longer survival period compared to males before the age of 55 (pre-menopausal period) (26). Further research is needed to clarify the extent to which these hormonal differences influence NPC outcomes. These findings emphasize the need for tailored prevention and control strategies that address these gender differences.

An unexpected finding of our study is that the incidence and mortality of NPC have been increasing significantly in people under 35 years of age in recent 10 years, which is an important finding of this study. Early-onset NPC may be strongly associated with genetic predispositions. Studies have identified certain HLA haplotypes (e.g., HLA-A*26, HLA-A*30, and HLA-DRB1*10) that increase the risk of NPC (27). Pathogenic heterozygous germline variants in MST1R, which encodes the macrophage-stimulating 1 receptor critical for host defense against viral infections, are strongly linked to the early onset of NPC (28). These genetic factors may predispose individuals to the disease at a younger age. Early exposure to EBV, combined with reactivation due to immune system dysregulation, may lead to early-onset NPC (29). Li et al.’s study revealed that patients with early-onset NPC exhibited higher EBV-DNA positivity rates compared to those with late-onset NPC (30). NPC is more common in southern China, characterized by a preference for consuming preserved foods (6). The interplay between genetic predispositions and environmental factors in these regions may account for the higher risk of early-onset cases. Population-based plasma EBV DNA testing for NPC screening helps in the detection of early-onset NPC (31). Furthermore, exposure to formaldehyde in occupational settings has been identified as a significant risk factor for nasopharyngeal carcinoma (9). Despite the Chinese government’s implementation of various standards to control formaldehyde exposure, levels in residences, office spaces, industrial facilities, public areas, and even food frequently surpass the established national limits (32). Individuals employed in industries such as resin production, textiles, leather processing, rubber manufacturing, cement, and plastics, as well as those working in anatomical or pathology laboratories, are considered to be at elevated risk of formaldehyde exposure due to the nature of their occupational environments (33). Environmental protection authorities should strengthen the enforcement of environmental laws and reduce formaldehyde emissions, which would help decrease the incidence of NPC.

This study found that NPC incidence peaks at ages 50–54 for both males and females, while mortality increases with age, peaking at 70–74 for males and 75–79 for females, consistent with Wu et al.’s findings (34). The high incidence of NPC observed in the 50–54 age group may reflect cumulative exposures, including smoking, from earlier life stages. As smoking is a known risk factor with a long latency period for cancer development, historical smoking behaviors in this cohort may partly explain the elevated incidence (23). Younger patients have higher overall survival and cancer-specific survival rates compared to older patients (30). Starting from the age of 65, the risk of cancer and related mortality significantly increase. Metabolic changes that occur with aging can create a systemic environment conducive to tumor progression and invasiveness (35). According to a survey conducted in Guangzhou, the infection rate of EBV increases with age: 7.07% in individuals aged 20-39, 7.86% in those aged 40-59, 10.11% in those aged 60-79, and 10.99% in individuals aged 80 and above (36). Aging accelerates the decline in immune function, accompanied by a reduction in lymphocyte and albumin levels (37). Combined with EBV positivity, this leads to a decreased overall survival in elderly patients (38). With China’s increasingly aging population and social transformation, the disease burden, including NPC, will continue to grow (34). This pattern highlights the importance of age-specific interventions, particularly screening and monitoring for middle-aged individuals and targeted treatments for the elderly, to reduce the burden of NPC.

The period effect showed an initial decline followed by stabilization, indicating that recent advancements in medical technologies and public health policies have had a positive impact (31). China ratified the Framework Convention on Tobacco Control in 2005, demonstrating the government’s heightened focus on tobacco control efforts (39). Improved public health awareness and screening initiatives have facilitated earlier case detection, resulting in better treatment outcomes and lower mortality rates (15). However, the continued stabilization of these effects suggests that further progress in reducing NPC burden may require more aggressive and innovative interventions, particularly in high-risk regions. The age-period-cohort analysis demonstrated that the cohort effect for NPC incidence and mortality has declined over time, suggesting that individuals born in more recent cohorts are at lower risk. This decline may reflect improvements in living conditions, dietary habits, and healthcare access over successive generations.

One of the strengths of our study is the use of data from the CCRAR, which provides a more accurate reflection of the true disease burden compared to global databases. However, several limitations should be noted. Firstly, the data coverage is limited to cancer surveillance sites that represent approximately 37-38% of the Chinese population. This may not fully capture the national burden, and the absence of geographic stratification restricts our ability to explore regional disparities in NPC incidence and mortality. Secondly, registry-based data are subject to potential reporting errors and misclassification biases, which could affect the accuracy of incidence and mortality estimates. Thirdly, while the APC model provides valuable insights into temporal trends, it cannot adjust for important confounding factors such as Epstein-Barr virus (EBV) infection status, lifestyle factors, and socioeconomic variations due to the aggregate nature of the data. Lastly, as an ecological study based on aggregated population-level data, this research cannot establish causality or infer associations at the individual level.

## Conclusions

Our findings confirm the effectiveness of existing NPC prevention and control strategies in China, as evidenced by the steady decline in incidence and mortality rates. Efforts to improve early detection, reduce exposure to risk factors, and enhance access to healthcare services will be essential for sustaining progress and reducing the burden of NPC in the future. Continued monitoring and evaluation of trends in NPC incidence and mortality are critical for guiding public health policies and optimizing resource allocation to combat this disease effectively.

## Data Availability

The raw data supporting the conclusions of this article will be made available by the authors, without undue reservation.
