# Supplementary material for: Trends in incidence and mortality of nasopharyngeal cancer in China (2004–2018): an age-period-cohort analysis
Source: Front Oncol. 2025 Jul 8;15:1592217. doi: 10.3389/fonc.2025.1592217 (PMC12279478; doi:10.3389/fonc.2025.1592217)
Supplement: Supplementary file 1 [file Table1.docx]

**Table S1** Wald Chi-square tests for estimable parameters in the APC model

| Metric | Null Hypothesis | Male | | Female | |
| --- | --- | --- | --- | --- | --- |
|  |  | Chi-square | P-value | Chi-square | P-value |
| Incidence | NetDrift = 0 | 39.91 | < 0.001 | 51.08 | < 0.001 |
|  | All Age Deviations = 0 | 552.06 | < 0.001 | 218.04 | < 0.001 |
|  | All Period RR = 1 | 73.35 | < 0.001 | 92.11 | < 0.001 |
|  | All Cohort RR = 1 | 83.80 | < 0.001 | 113.16 | < 0.001 |
|  | All Local Drifts = Net Drift | 31.00 | < 0.01 | 42.13 | < 0.001 |
| Death | NetDrift = 0 | 48.30 | < 0.001 | 59.95 | < 0.001 |
|  | All Age Deviations = 0 | 150.37 | < 0.001 | 167.82 | < 0.001 |
|  | All Period RR = 1 | 81.71 | < 0.001 | 82.30 | < 0.001 |
|  | All Cohort RR = 1 | 96.12 | < 0.001 | 192.79 | < 0.001 |
|  | All Local Drifts = Net Drift | 19.32 | 0.15 | 45.31 | < 0.001 |
